# An Automated Phenotype-Driven Approach (*Gene*Force) for Refining Metabolic and Regulatory Models

**DOI:** 10.1371/journal.pcbi.1000970

**Published:** 2010-10-28

**Authors:** Dipak Barua, Joonhoon Kim, Jennifer L. Reed

**Affiliations:** 1 Department of Chemical and Biological Engineering, University of Wisconsin-Madison, Madison, Wisconsin, United States of America; 2 DOE Great Lakes Bioenergy Research Center, University of Wisconsin-Madison, Madison, Wisconsin, United States of America; The Pennsylvania State University, United States of America

## Abstract

Integrated constraint-based metabolic and regulatory models can accurately predict cellular growth phenotypes arising from genetic and environmental perturbations. Challenges in constructing such models involve the limited availability of information about transcription factor—gene target interactions and computational methods to quickly refine models based on additional datasets. In this study, we developed an algorithm, *Gene*Force, to identify incorrect regulatory rules and gene-protein-reaction associations in integrated metabolic and regulatory models. We applied the algorithm to refine integrated models of *Escherichia coli* and *Salmonella typhimurium*, and experimentally validated some of the algorithm's suggested refinements. The adjusted *E. coli* model showed improved accuracy (∼80.0%) for predicting growth phenotypes for 50,557 cases (knockout mutants tested for growth in different environmental conditions). In addition to identifying needed model corrections, the algorithm was used to identify native *E. coli* genes that, if over-expressed, would allow *E. coli* to grow in new environments. We envision that this approach will enable the rapid development and assessment of genome-scale metabolic and regulatory network models for less characterized organisms, as such models can be constructed from genome annotations and *cis*-regulatory network predictions.

## Author Summary

Computational models of biological networks are useful for explaining experimental observations and predicting phenotypic behaviors. The construction of genome-scale metabolic and regulatory models is still a labor-intensive process, even with the availability of genome sequences and high-throughput datasets. Since our knowledge about biological systems is incomplete, these models are iteratively refined and validated as we discover new connections in biological networks, and eliminate inconsistencies between model predictions and experimental observations. To enable researchers to quickly determine what causes discrepancies between observed phenotypes and model predictions, we developed a new approach (*Gene*Force) that automatically corrects integrated metabolic and transcriptional regulatory network models. To illustrate the utility of the approach, we applied the developed method to well-curated models of *E. coli* metabolism and regulation. We found that the approach significantly improved the accuracy of phenotype predictions and suggested changes needed to the metabolic and/or regulatory models. We also used the approach to identify rescue non-growth phenotypes and to evaluate the conservation of transcriptional regulatory interactions between *E. coli* and *S. typhimurium*. The developed approach helps reconcile discrepancies between model predictions and experimental data by hypothesizing required network changes, and helps facilitate the development of new genome-scale models.

## Introduction

A current challenge in systems biology is reconstructing transcriptional regulatory networks from experimental data (e.g. gene expression, genome sequence, and DNA-protein interaction), due to the complexity of interactions in these networks and the limited information on network components and interactions for most organisms [1], [2]. Even for well-studied model organisms, such as *E. coli* and *Saccharomyces cerevisiae*, inferred or indirect regulatory interactions have to be included in genome-scale transcriptional regulatory reconstructions due to existing knowledge gaps in how genes are transcriptionally regulated [3], [4]. Reconstructed regulatory networks will be incomplete, reflecting incomplete knowledge about *cis*-regulatory networks, and may include incorrect interactions. As such, methods for iterative validation and refinement of regulatory reconstructions are needed in order to assess new experimental datasets as they emerge [5], [6]. Such approaches need to identify and eliminate inconsistencies between the reconstructed network and new experimental data, and to include newly discovered network interactions [3]. However, identifying the cause of inconsistencies in a highly interconnected network using manual efforts is not a trivial task, and can be labor intensive particularly for genome–scale transcriptional regulatory network models. Therefore, systematic approaches that automate this iterative procedure are useful for identifying new or incorrect connections in biological models; such approaches have been developed for analysis and correction of metabolic networks [7]–[9]. In this paper, we present an approach that allows for the automated adjustment of an integrated genome-scale metabolic and transcriptional regulatory network model, by comparing the emergent properties of the integrated networks to cellular growth phenotypes. These adjustments result in testable hypotheses about transcriptional regulation and metabolism in organisms.

While there are many types of regulatory modeling approaches (reviewed in [10]), Boolean modeling of regulatory interactions can be beneficial when modeling large-scale regulatory networks because (i) such formalism requires minimal parametric details to be incorporated [11], and (ii) these Boolean models can be integrated with constraint-based metabolic models [3], [4]. One of the commonly used constraint-based modeling approaches for metabolic models is flux balance analysis (FBA), which predicts an optimal steady-state flux distribution in a metabolic network [12]. This can be extended to integrated metabolic and regulatory models, referred to as regulated flux balance analysis (rFBA), which accounts for transcriptional regulation as well as the other governing physicochemical constraints [13], [14]. While the metabolic and regulatory models in rFBA are solved iteratively, newer approaches for integrating metabolic and regulatory models allow the models to be combined into a single model using an mixed-integer linear programming (MILP) formalism [15]. In this case, steady-state regulatory flux balance analysis (SR-FBA) is used to identify optimal flux distributions that satisfy both models simultaneously. We have recently developed an efficient SR-FBA formulation that systematically integrates transcriptional regulatory and metabolic networks [16] which was used here.

In this work, we developed an algorithm (called *Gene*Force) to reconcile integrated regulatory and metabolic model predictions with experimental data, by automatically identifying and overriding transcriptional regulatory rules that cause inconsistencies between model predictions and experimental observations. The approach can be used in cases where both the experimental data and an un-regulated metabolic model agree on a positive growth phenotype (cells can grow), but the integrated metabolic and regulatory model predicts a non-growth phenotype (cells cannot grow). In these cases, the *Gene*Force algorithm allows the integrated metabolic and regulatory model to achieve growth by violating regulatory rules as needed, while minimizing the total number of regulatory violations in order to maximally preserve the original regulatory interactions present in the regulatory network reconstruction. These rule violations indicate that regulatory rules describing gene expression are incorrect or that isozymes or alternative pathways are present in the metabolic network.

We first applied the *Gene*Force method to refine the genome-scale transcriptional regulatory network for *E. coli*, *i*MC1010^v1^ [3] which was updated here to include newly discovered Lrp regulatory interactions [17]. The algorithm was used to analyze a large collection of ∼50,000 *E. coli* knockout mutant growth phenotypes [18], [19], and the suggested regulatory corrections resulted in a ∼1–8% improvement in model accuracy over the original models, which had already been adjusted during their initial development to improve model accuracy. In addition to correcting regulatory rules, we applied the *Gene*Force algorithm to predict genes that, if overexpressed or constitutively expressed, could rescue non-growth phenotypes of *E. coli* strains (wild-type or mutants) in certain growth environments. Finally, we applied the *Gene*Force method to investigate the conservation of transcriptional regulatory interactions between *E. coli* and *S. typhimurium*. The *E. coli* transcriptional regulatory rules were integrated with a metabolic model for *S. typhimurium* that included metabolic genes and reactions from a recent metabolic reconstruction *i*RR1083 [20]. *Gene*Force suggested a small set of rule corrections for this hybrid network model were needed, based on analysis of *S. typhimurium* growth phenotyping data, suggesting that regulation may be highly conserved between these two organisms. While the approach has been used here to correct Boolean representations of transcriptional regulation, it could easily be extended to consider non-Boolean approaches to modeling transcriptional regulation as they are developed.

## Results

### Regulatory rule correction algorithm, *Gene*Force

We developed an automated MILP approach, *Gene*Force, to identify problematic Boolean regulatory rules in an integrated metabolic and transcriptional regulatory model. The method identifies regulatory rules that prevent the models from predicting cellular growth in conditions, which are capable of supporting growth experimentally. The approach can be used when the integrated metabolic and regulatory model does not predict growth, but experimental data and metabolic model predictions (without any regulatory constraints) indicate growth occurs.

The basic idea of the *Gene*Force algorithm is to allow the integrated metabolic and regulatory model to violate a minimal set of transcriptional regulatory rules so that growth can occur in a particular condition. The algorithm therefore adds an additional constraint that the model must satisfy a minimal threshold growth rate. The algorithm uses a set of ‘rule-violation’ equations (see Supporting Information Text S1 for details) to relax certain regulatory constraints (by allowing for expression of un-expressed genes) thus allowing the model to readjust the metabolic and regulatory model solution space to include solutions with growth rates exceeding the minimum threshold. The ‘rule violation’ equations, invoked at the gene level, allow the regulatory rules for metabolic genes to be violated using additional surrogate gene expression indicators (y′_g_) that can differ in value from the gene expression indicators (y_g_), the latter of which are determined by Boolean regulatory rules. Normally in the integrated metabolic and regulatory model a flux is constrained to be zero if the necessary metabolic genes are determined to be un-expressed (y_g_ = 0). In the *Gene*Force algorithm, the bounds on the metabolic fluxes are dependent on y′_g_ instead of y_g_. The reaction dependence on y′_g_ then allows the model to override a minimum number of gene expression indicators (where y_g_ = 0 but y′_g_ = 1) so that the threshold growth rate can be achieved.

**Figure 1:**
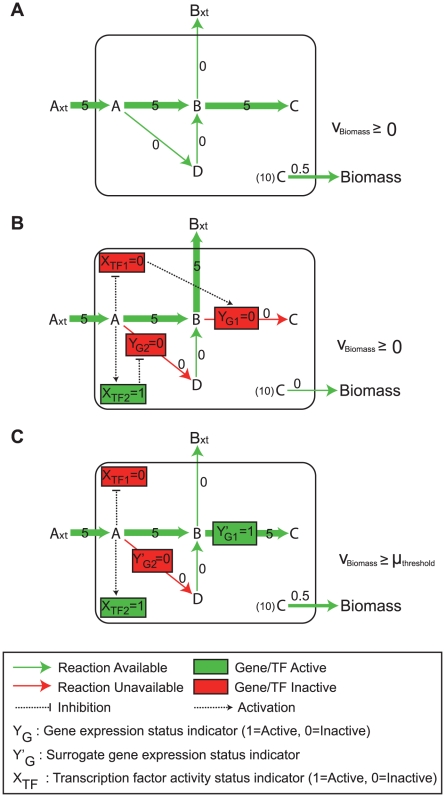
Example Network Illustrating the *Gen*eForce Approach. (**A**) Predicted fluxes through an un-regulated metabolic network, where all reactions are available (indicated by the green arrow) and flux through the biomass reaction (v_Biomass_) is maximized. The numbers and thickness of the arrows indicate flux values. (**B**) Predicted flux through an integrated metabolic and regulatory model (SR-FBA), where numbers and arrow thicknesses indicate flux values. The regulatory network includes regulation of two genes (G1 and G2) by two transcription factors (TF1 and TF2), where TF1 activates G1 and TF2 represses G2. G1 is needed for the B→C reaction and G2 is needed for the A→D reaction. Binary gene expression status (y_G1_ and y_G2_) and transcription factor activity (x_TF1_ and x_TF2_) indicators show the expression and binding status of G1, G2, TF1 and TF2, respectively, with value 1 indicating the expressed/active condition and 0 indicating the unexpressed/inactive condition. Regulatory interactions are shown as dashed lines, where a normal or blunt arrowhead indicates activation and repression, respectively. The colors indicate the state (active = green, inactive = red) of transcription factors and metabolic gene expression, or the availability of metabolic reactions (available = green, unavailable = red). (**C**) Fluxes and surrogate gene expression indicator values as predicted by the *Gene*Force approach. The reactions (B→C and A→D) are now dependent on the surrogate gene expression indicators (y′_G1_ and y′_G2_) instead of the expression status of genes G1 and G2 (y_G1_ and y_G2_). A threshold biomass flux (μ_threshold_) is set as a constraint and the *Gene*Force algorithm minimizes the sum of the differences between the surrogate gene expression indicators (shown in c) and the gene expression indicators (shown in b) while satisfying this constraint.

The example in Figure 1 illustrates how the *Gene*Force algorithm uses the rule violation technique to achieve non-zero growth in an integrated metabolic and regulatory model in agreement with the metabolic model predictions. As shown in the Figure 1A the metabolic model predicts positive growth in the presence of A_xt_, whereas the integrated metabolic and regulatory model predicts no growth due to the regulatory interactions between gene G1 and transcription factor TF1 (Figure 1B). Expression of G1 is needed for growth since the corresponding enzyme catalyzes an essential reaction (B→C), but expression of G1 requires TF1 to be active, and the binding activity of TF1 is inhibited by metabolite A. This non-growth phenotype is overcome in the *Gene*Force algorithm by making the reaction availability dependent on the surrogate gene expression indicator y′_G1_, which is not dependent on the regulatory rules. In this case, y_G1_ = 0 and y′_G1_ = 1 since the associated reaction is essential for growth (Figure 1C). Since the flux ranges in *Gene*Force are directly dependent on y′_g_ rather than y_g_, the reaction associated with G1 can carry flux through it allowing growth to occur, even though the gene is not expressed in the Boolean model. It should be noted that the other regulatory rule in the network (y_G2_ repressed by TF2) was not overruled (y_G2_′ = y_G2_ = 0), even though y_G2_′ could also take the value 1 instead of 0. This is because the algorithm minimizes the sum of the distances between the surrogate and the original gene expression indicators, (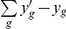), and hence the number of regulatory rule violations by the algorithm. Minimization of this objective function forces the binary vector of y′_g_ to remain as close as possible to that of y_g_, thus minimizing the number of rule violations. This ensures that the original, literature-derived regulatory rules are maximally conserved and reflected in the predicted behavior of the integrated metabolic and regulatory network.

### Rule correction by *Gene*Force: application to an *E. coli* regulatory network model

We used the *Gene*Force algorithm to refine the regulatory rules in an updated metabolic and regulatory *E. coli* model based on *i*MC104 (the regulatory portion of the integrated *i*MC1010^v1^ model [3]), where we had revised the regulatory rules for genes in the Lrp regulon based on experimental data [17] (see methods for details). The Lrp-modified *i*MC104 model was combined with a metabolic model and the resulting integrated model was used to predict growth phenotypes that were compared to experimental growth phenotypes for a large number of knockout mutants tested for growth in various conditions [18], [19]. The model refinements were carried out in three successive steps. First, the updated regulatory rules from *i*MC104 were integrated with the metabolic model *i*JR904 [21] and rule corrections were made to give the first refined version of the regulatory model *i*MC105A. Second, the *i*MC105A regulatory model was integrated with an updated metabolic network *i*AF1260 [22] and adjusted to give the second refined regulatory model, *i*MC105AB. Finally, the *i*MC105AB regulatory model was further refined using phenotypic data generated in this study for three global transcription factor knockout mutants (Δ*arcA*, Δ*purR* and Δ*lrp*) to give the final version of the regulatory model *i*MC105*ABC*. Here we consider the regulatory models (*i*MC104, *i*MC105A, *i*MC105AB, and *i*MC105ABC) to be just the regulatory part of the integrated models (the number indicates the total number of transcription factors).

**Figure 2:**
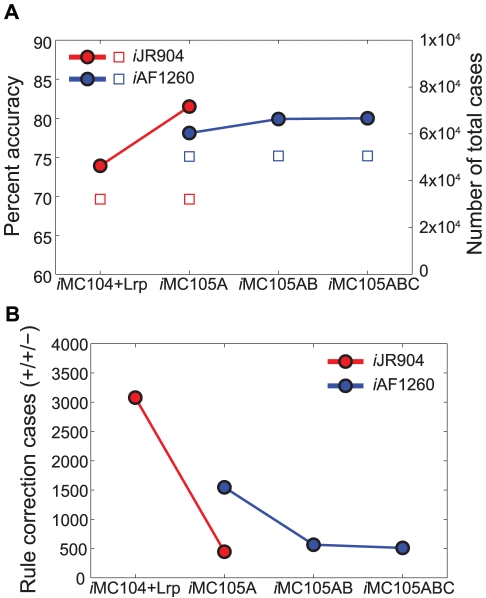
Accuracy and Number of Rule Correction Cases. Application of *Gene*Force to correct growth phenotype predictions by overriding regulatory rules (**A**) Growth phenotype prediction accuracy of integrated regulatory-metabolic network models at various steps of regulatory network refinement. Accuracy (solid circles) is calculated by dividing total number of correct (experimentally consistent) predictions by the total number of cases evaluated (open squares) at each step. The colors correspond to the metabolic networks used in the integrated metabolic and regulatory network models with red for *i*JR904 and blue for *i*AF1260. (**B**) The total number of ‘rule correction’ cases (solid circles) for each regulatory network is plotted. Such cases are represented by +/+/− (Exp/Met/Met+Reg) in the growth comparison tables (Supporting Information Table S1 and S2).

Integration of *i*JR904 with Lrp-modified *i*MC104 regulatory rules allowed comparison of 32,050 growth phenotype predictions to experimental data (Supporting Information Table S1). The *Gene*Force algorithm identified genes with possible problematic regulatory rules in 3,079 out of the 32,050 cases examined, where each case represents a mutant grown in a different condition. Alternative optimal solutions exist for only 298 of the 3,079 cases, where most (281 out of 298) were needed to correct predictions for growth on L-serine as a nitrogen source or the Δ*sdaB* mutant. These 3,079 cases correspond to cases where a zero growth prediction by the integrated metabolic and regulatory model contradicted both the experimental data and the metabolic model prediction (+/+/−; where + indicates growth and − indicates no growth, and the order corresponds to the results from experiments / metabolic model / integrated model). Not all regulatory rules identified by the algorithm in the 3,079 cases were adjusted, as they may cause new incorrect predictions in other conditions. Instead, corrections were made for regulatory rules that were frequently identified as problematic for a particular knockout mutant or growth environment (Table 1, refinement step A). In total, regulatory rules for ten genes (*glmU*, *ilvY*, *ilvC*, *sdaC*, *cycA*, *gcvB*, *dsdX*, *rpiR*, *acnA*, and *ilvA*) were corrected in the first regulatory model refinement, *i*MC105A. Two gene-protein-reaction (GPR) associations were also corrected in the metabolic model for two amino acid transport reactions (L-methionine and D-serine). These model adjustments led to an ∼8% improvement in the overall accuracy of the integrated model from 73.9% to 81.5% (Table 2 and Figure 2A). Due to the addition of the transcriptional regulator *gcvB*, this revised regulatory network *i*MC105A contained a total of 105 transcription factors.

**Table 1:** *E. coli* model refinements and the conditions under which they were identified by *Gene*Force.

Refinement Step	Gene	Original Rule	Refined Rule	Condition#	Comment
**A**	*metINQ*	(NOT MetJ)	GPR correction	Gly-Met (N)Met-Ala (N)	Unknown transporter for L-methionine (PMID: 4604763)
**A**	*glmU*	(NagC)	(ON)	N-acetyl-D-glucosamine (C,N)N-acetyl-D-mannosamine (C,N)N-acetyl-neuraminic acid (N)	Essential gene (PMID: 8407787)
**A**	*ilvY*	(NOT val-L(e)>0 )	(ON)	b3773 (*ilvY*)	á-acetolactate or á-acetohydroxybutyrate inducer for *ilvY* (PMID: 10588699)
**A**	*ilvC*	(ilvY)	(ilvY AND NOT (val-L(e)>0)) OR (NOT ilvY)	b3773 (*ilvY*)	Constitutive expression of *ilvC* in *ilvY* strain (PMID: 6783625)
**A**	*sdaC* *	(Crp AND (NOT Lrp OR (leu-L(e)>0)))	((Crp AND (NOT Lrp OR (leu-L(e)>0)))) OR (ser-L(e)>0)	L-serine (N)	Transporters for ser-L; *sdaC* ser-L specific, *sstT* major, *tdcC* anaerobic (PMID: 8026499)
**A**	*cycA*	(NOT Lrp OR (leu-L(e)>0))	(NOT GcvB)	D-alanine (C,N)	No Lrp binding; CycA transporter for 6 amino acids (PMID: 19118351)
**A**	*gcvB*		(NOT GcvR AND GcvA)	D-alanine (C,N)	New regulatory small RNA (PMID: 10972807)
**A**	*dsdX*		GPR correction DsdC or (DsdC and Crp)	D-serine (C,N)	New ser-D transporter (This study, PMID: 16952954); regulation (PMID: 7592420)
**A**	*rpiR*	(NOT (rib-D(e)>0))	(NOT ((all-D(e)>0) OR (rib-D(e)>0)))	b2914 (*rpiA*)	*i*JR904 requires *rpiB* for *rpiA* strain (PMID: 10559180)
**A**	*acnA*	(SoxS)	(ON)	b0118 (*acnB*)	Two aconitases (PMID: 9202458)
**A**	*ilvA* *	(NOT Lrp OR (leu-L(e)>0))	(ON)	b2797 (*sdaB*)	L-serine/L-threonine deaminases; SdaA (anaerobic), TdcB (anaerobic), IlvA (PMID: 13405870, 15155761)
**B**	*argD*	(NOT ArgR)	(ON)	L-arginine (N)	Required for L-lysine biosynthesis
**B**	*astABCDE*	((NOT(Growth>0) AND RpoS) OR (NRI_hi AND RpoN)) AND (NOT Lrp OR (leu-L(e)>0))	((NOT(Growth>0) AND RpoS) OR (NRI_hi AND RpoN))	L-arginine (N)	AST pathway for L-arginine degradation (PMID: 9696779)
**B**	*speA*	(NOT (PurR))	(NOT (PurR)) AND (NOT (AGMT>0))	L-arginine (C)	Putrescine inhibits transcription of *speA* (PMID: 1646785)
**B**	*metH* *	(MetR)	(metR) OR (met-L(e)>0)	Gly-Met (N)Met-Ala (N)	methionine represses *metE*, but not *metH* (PMID: 16622061)
**B**	*thrA*	(NOT (thr-L(e)>0 OR ile-L(e)>0)) AND (NOT Lrp OR (leu-L(e)>0))	(NOT (thr-L(e)>0 OR ile-L(e)>0))	Gly-Met (N)Met-Ala (N)	methionine represses *metL*, but not *thrA* (PMID: 3910040)
**B**	*rhaS*	(RhaR)	(RhaR OR (RhaR AND Crp))	L-lyxose (C)	*rhaA*, *rhaT* <- *RhaS* <- *RhaR/emph> (PMID: 8757746)*
**B**	*rhaR*	(rmn(e)>0)	(rmn(e)>0 OR lyx(e)>0 OR man(e))	L-lyxose (C)	*RhaR* <- rhamnose, lyxose, mannose (PMID: 8757746)
**C**	*serC*	(Lrp AND NOT (leu-L(e)>0) OR (NOT (Crp)))	(ON)	b0889 (lrp)	Essential in glucose and glycerol minimal medium (PMID: 17012394)
**C**	*aroA*	(Lrp AND NOT (leu-L(e)>0))	(ON)	b0889 (lrp)	Essential in glucose and glycerol minimal medium (PMID: 17012394)
**C**	*leuD*	(NOT(leu-L(e)>0) AND Lrp)	(NOT(leu-L(e)>0)	b0889 (lrp)	Essential in glucose and glycerol minimal medium (PMID: 17012394)
**C**	*leuC*	(NOT(leu-L(e)>0) AND Lrp)	(NOT(leu-L(e)>0)	b0889 (lrp)	Essential in glucose and glycerol minimal medium (PMID: 17012394)
**C**	*leuB*	(NOT(leu-L(e)>0) AND Lrp)	(NOT(leu-L(e)>0)	b0889 (lrp)	Essential in glucose and glycerol minimal medium (PMID: 17012394)
**C**	*leuA*	(NOT(leu-L(e)>0) AND Lrp)	(NOT(leu-L(e)>0)	b0889 (lrp)	Essential in glucose and glycerol minimal medium (PMID: 17012394)
**C**	*ilvB* *	(NOT(leu-L(e)>0 OR val-L(e)>0) AND Crp) to (ON)	(ON)	b0889 (lrp)glucose (C)gluconate (C)	*ilvB* required in glucose condition (this study)
**C**	*ilvN* *	(NOT(leu-L(e)>0 OR val-L(e)>0) AND Crp) to (ON)	(ON)	b0889 (lrp)glucose (C)gluconate (C)	regulatory subunit of *ilvBN* encoded enzyme complex (PMID: 1512191)
**C**	*dctA* *	(((“CRP noMAN”) AND NOT(ArcA) AND (DcuR))	(ON)	b0889 (lrp)L-malate (C)	*dctA* deletion causes prolonged lag phase (this study)

*indicates alternative optimal solutions exist for this change.

# (C) indicates carbon source and (N) indicates nitrogen source.

**A**- Rule corrections needed for *i*MC104+*i*JR904.

**B**- Rule corrections needed for *i*MC105A+*i*AF1260.

**C-** Rule corrections needed for *i*MC105AB+*i*AF1260.

**Table 2:** Accuracy and number of rule correction and rescue non-growth cases at successive stages of regulatory rule refinements.

**Metabolic network** a	*i*JR904	*i*JR904	*i*AF1260	*i*AF1260	*i*AF1260
**Regulatory network** b	*i*MC104	*i*MC105A	*i*MC105A	*i*MC105AB	*i*MC105ABC
**Total comparisons** c	32,050	32,050	50,327	50,557	50,557
**Rule correction cases (+/+/−)**	3,079	445	1,546	565	510
**Rescue cases (−/+/−)**	2,041	1,847	2,130	2,087	2,070
**Integrated model accuracy** d	23,670 (73.9%)	26,112 (81.5%)	39,288 (78.1%)	40,403 (79.9%)	40,441 (80.0%)

a,b Metabolic and regulatory networks used in the integrated models.

c Total number of growth phenotypes analyzed.

d Number (percent) of cases where the integrated model predictions were in agreement with experimental data.

The second set of refinements (refinement step B), occurred when the *i*JR904 metabolic network was replaced with the updated metabolic network *i*AF1260 [22]. The inclusion of the latest metabolic network allowed integrated model predictions to be compared against 50,327 growth phenotypes, since more genes and environments are represented in this larger metabolic network (Supporting Information Table S2). Using this extended set of growth phenotypes, the algorithm identified a new set of problematic regulatory rules in *i*MC105A, and corrections were made for eleven additional genes, *argD*, *astCADBE*, *speA*, *metH*, *thrA*, *rhaS*, and *rhaR* (Table 1, refinement step B) leading to a second revision of the regulatory model, *i*MC105AB. Initial correction of the regulatory rule for *argD* fixed 262 errors (+/+/− changed to +/+/+) associated with cases where arginine is the nitrogen source, but also introduced 297 new errors (−/+/− changed to −/+/+) for cases where arginine is the carbon source. To correct these new errors we subsequently refined the rules for the *astCADBE* operon and *speA* gene, in addition to *argD*, to reconcile the model with both arginine conditions. The prediction accuracy of the integrated metabolic and regulatory model (*i*AF1260+*i*MC105A) was 78.1% before all eleven rule corrections were made, and with this additional second set of regulatory refinements, the *i*MC105AB model could achieve a slightly higher accuracy, 79.9% (Table 2 and Figure 2A) and with significantly greater coverage of the available experimental data (50,327 cases versus 32,050 cases).

In the third set of refinements, the refined regulatory model, *i*MC105AB, was tested by comparing predictions to newly acquired experimental data for three transcription factor knockout mutants (Δ*arcA*, *ΔpurR*, and Δ*lrp*). The five transcription factors in *i*MC105AB with the most metabolic gene targets are Crp, Fnr, ArcA, PurR and Lrp. Experimental data was already available for knockout mutants for two of these transcription factors (Crp and Fnr), however, growth phenotyping data for Δ*arcA*, Δ*purR*, and Δ*lrp* mutants was not available. Therefore, growth experiments on phenotype microarrays (Biolog, Hayward, CA) were conducted for the three mutant strains Δ*arcA*, Δ*purR*, and Δ*lrp*. The *Gene*Force algorithm identified an additional nine genes needing regulatory rule corrections (Table 1, refinement step C) which all caused incorrect phenotype predictions for the Δ*lrp* mutant (Supporting Information Table S3). Incorporation of these Lrp specific corrections led to the final refined version *i*MC105ABC, which resulted in a small overall improvement (0.01%) in model accuracy over the previous version *i*MC105AB (Table 2 and Figure 2A) when evaluating all data, but a large improvement for the new phenotype measurements of the three transcription factor deletion mutants (81.7% accuracy using *i*MC105ABC vs. 66.1% using *i*MC105AB for Δ*lrp*, Δ*purR*, and Δ*arcA* phenotypes).

**Figure 3:**
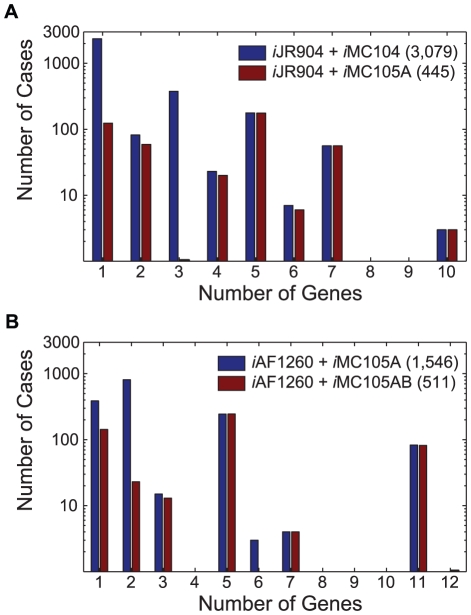
Number of Rule Corrections Needed to Correct Model Predictions. Distribution of rule corrections for +/+/− cases before and after rule corrections for (**A**) *i*JR904 with rules from *i*MC104 (with Lrp modified regulatory rules) and *i*MC105A, and (**B**) *i*AF1260 with rules from *i*MC105A and *i*MC105AB. The total number of +/+/− cases for each integrated model is indicated in parenthesis in the legend. For each +/+/− case the minimum number of genes requiring regulatory rule corrections was determined. Panels **A** and **B** are histograms representing the number of cases where 1, 2, 3, 4, 5, 6, 7, 8, 9, 10, 11, or 12 genes need regulatory rule corrections.

For each +/+/− case the minimum number of genes whose regulatory rules had been violated by the *Gene*Force algorithm was determined. The distribution of the number of rule corrections needed for the +/+/− cases is shown for the first two refinement steps in Figure 3 before and after the model adjustments were made (listed in Table 1). The results show that in most cases a single regulatory rule was preventing the integrated model from making the correct prediction. The first set of refinements eliminated most of the +/+/− cases (Figure 3A), leaving fewer genes needing rule corrections in the subsequent steps (Figure 3B), even though more experimental data (50,557 versus 32,050) could be compared to model predictions.

Alternative optimal solutions were generated for each refinement step by adding integer-cut constraints and re-solving the *Gene*Force problem. The number of +/+/− cases for which alternative optimal solutions exist can be found in Supporting Information Table S4. In most cases, they were specific to a particular knockout mutant or growth environment, and the alternative optimal solutions were two or three isozymes catalyzing an essential reaction. For some instances we were able to find enough information in the literature to determine the most likely isozymes involved (*ilvA*, *metH*, *sdaC* and *thrA*). For other cases described below (*dctA*, *rpiB*, and *ilvBN*), we performed additional growth phenotyping experiments to determine the final set of corrections. Overall application of the *Gene*Force algorithm to correct +/+/− cases led to (i) changes in the regulatory rules for metabolic genes (e.g. *glmU*, *ilvBN*, and *dctA*) (ii) changes in the rules for TF activities (e.g. RpiR), or (iii) changes in the gene-protein-reaction (GPR) associations in the metabolic network (e.g. *dsdX*). Some examples from the different types of changes are presented below, and in some cases additional mutant phenotypes were screened by experiments to confirm the necessary model changes identified by *Gene*Force.

In our analysis, the regulatory rule describing the regulation of *glmU* by the NagC transcription factor was identified as the most problematic rule, causing approximately one third of the total incorrect zero growth predictions by the Lrp-modified *i*MC104+*i*JR904 integrated model. *Gene*Force identified the regulatory rule for *glmU* gene as needing a correction for most mutants grown in conditions where any of the three amino sugars, N-acetylglucosamine (GlcNAc), N-acetylneuraminate, and N-acetylmannosamine were present. GlmU catalyzes two consecutive reactions producing an essential precursor UDP-N-acetyl-glucosamine (UDP-GlcNAc) for the cell wall of *E. coli* [23], [24]. This gene has been found to be essential in *E. coli* [25], supporting *Gene*Force's prediction that the regulatory rule for *glmU* is incorrect.

The following two regulatory rules in *i*MC104 precluded the gene from being expressed in the integrated model under certain conditions: ‘NagC is active if NOT (GlcNAc OR glucosamine-6-phosphate)’ and ‘*glmU* is expressed if NagC is active’. The first rule prohibited NagC from being active in the presence of any of the three amino sugars because glucosamine-6-phosphate is a common intermediate in their degradation pathways. The inactivity of NagC subsequently prohibited the expression of *glmU* in the integrated model, resulting in a non-growth phenotype prediction. *Gene*Force violated the *glmU* regulatory rule so that GlmU can carry out the two essential reactions. Although the regulatory rules shown above were in agreement with experimental data reported in the literature, the Boolean representation of the regulatory interaction was too stringent in the model. The *glmU* gene contains two upstream promoters P1 and P2, and the transcription factor NagC is shown to induce expression using the promoter P1 in the absence of any of the three amino sugars [26]. However, the second *glmU* promoter, P2, is weakly induced in presence of N-acetylglucosamine, suggesting that the activating role of NagC could be dispensable for this promoter [26]. This suggests that the expression of *glmU* is not completely abolished when NagC is inactive, and that the low level of induction at P2 is still sufficient to allow for the production of UDP-GlcNAc. Since *glmU* is required for growth in other environments as well, it is always expressed in the refined set of regulatory rules.

**Figure 4:**
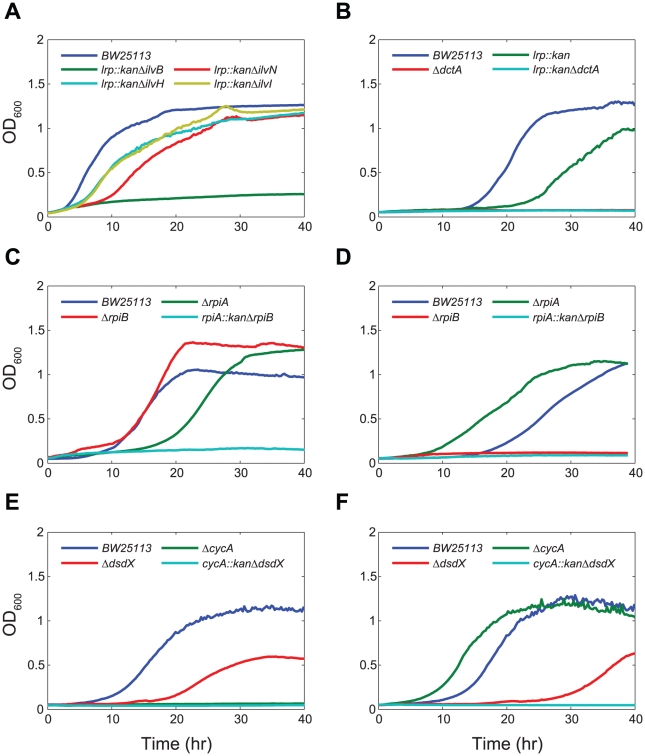
Phenotyping Experiments to Confirm Rule Corrections. Growth phenotype screens for (**A**) BW25113 (parent strain), *lrp::kan* Δ*ilvB*, *lrp::kan* Δ*ilvN*, *lrp::kan* Δ*ilvH*, and *lrp::kan* Δ*ilvI* on glucose M9 minimal media, (**B**) BW25113, *lrp::kan*, Δ*dctA*, and *lrp::kan* Δ*dctA* on L-malate M9 minimal media, (**C**) BW25113, Δ*rpiA*, Δ*rpiB*, and *rpiA::kan* Δ*rpiB* on D-ribose M9 minimal media, (**D**) BW25113, Δ*rpiA*, Δ*rpiB*, and *rpiA::kan* Δ*rpiB* on D-allose M9 minimal media, (**E**) BW25113, Δ*cycA*, Δ*dsdX*, and *cycA::kan* Δ*dsdX* on D-alanine M9 minimal media, and (**F**) BW25113, Δ*cycA*, Δ*dsdX*, and *cycA::kan* Δ*dsdX* on D-serine M9 minimal media.

The integrated model made incorrect predictions for the Δ*lrp* mutant in a few different conditions, including growth on glucose, gluconate, and L-malate as sole carbon sources. For the glucose and gluconate conditions, *Gene*Force found that either *ilvHI* or *ilvBN* needed to be expressed since these two isozymes are used for the synthesis of branched chain amino acids. To evaluate which of these isozymes is used by the cells, we screened a number of Lrp double mutants for growth on glucose and found that only Δ*lrp*Δ*ilvB* is unable to grow; however, Δ*lrp*Δ*ilvN*, Δ*lrp*Δ*ilvH*, and Δ*lrp*Δ*ilvI* were all capable of growing in glucose minimal media (Figure 4A). This is consistent with earlier reports that the catalytic subunits (*ilvB* and *ilvI*) are still active in the absence of the smaller regulatory subunits (*ilvM*, *ilvN* and *ilvH*) [27]. To reconcile the positive growth phenotype of a Δ*lrp* mutant grown on malate, *Gene*Force needed to override the regulatory rule for one of the malate transporters in *E. coli*. We subsequently found that Δ*dctA* and Δ*lrp*Δ*dctA* mutants did not grow on L-malate, while the Δ*lrp* mutant grew (Figure 4B) , implying that the *dctA* rule needed correction.

Ribose-5-phosphate isomerase (RPI) catalyzes the reversible conversion of ribose-5-phosphate to ribulose-5-phosphate in the pentose phosphate pathway. Two RPIs have been identified in *E. coli*, RpiA and RpiB, which are genetically and biochemically distinct. RpiA is constitutively expressed and accounts for most of the RPI activity in wild-type cells [28]. RpiB also functions as an allose-6-phosphate isomerase, catalyzing the second step in the allose degradation pathway [29]. It has been shown that *rpiB* expression is repressed by a regulator, RpiR, which is located on the same operon [30]. We subsequently measured growth of Δ*rpiA*, Δ*rpiB*, and Δ*rpiA*Δ*rpiB* mutants on D-ribose and D-allose, and found that only the double deletion exhibited a lethal phenotype on D-ribose (Figure 4C), while neither Δ*rpiB* nor Δ*rpiA*Δ*rpiB* mutant grew on D-allose (Figure 4D). The original regulatory rule for RpiR had only D-ribose as an inducer; as a result no growth in the D-allose medium condition was incorrectly predicted by the integrated model. We subsequently changed the rule for *rpiR* to also include D-allose as an inducer based on the study of allose catabolism [31].

Another interesting case was the suggested regulatory refinements for *cycA* based on the utilization of D-alanine and D-serine. In this case the algorithm helped lead to improvements in GPR associations in the metabolic network as well as the regulatory rule for *cycA*. The integrated model incorrectly predicted that these compounds could not be used as carbon and nitrogen sources, which *Gene*Force attributed to the expression rule for the CycA transporter. We subsequently measured growth of Δ*cycA*, Δ*dsdX*, and Δ*cycA*Δ*dsdX* mutants, and found that the Δ*cycA* and Δ*cycA*Δ*dsdX* mutant were unable to grow with D-alanine as a carbon source (Figure 4E) and that only the Δ*cycA*Δ*dsdX* double mutant was unable to grow with D-serine as a carbon source (Figure 4F). This indicates that *cycA* is expressed under both conditions and that *dsdX* is also expressed when D-serine is present as a carbon source. The transport of D-serine by DsdX has only been shown in an uropathogenic strain of *E. coli* [32] and based on our phenotyping results this protein appears to have the same function in BW25113 as well. As a result, the DsdX transporter was to be added to the metabolic model and the regulatory rule for *cycA* was modified. Altogether, these experimental results illustrate how *Gene*Force can help identify incorrect regulatory rules or missing metabolic functionality which cause model-data discrepancies.

To investigate the effects the model corrections have in other conditions we evaluated how many new false positives were introduced (i.e. −/+/− cases became −/+/+) for each refinement step (Supporting Information Table S4) and whether the predicted flux distributions would change using flux variability analysis [33]. In refinement steps A and B, the number of new false positives was only ∼7% of the total number of corrected errors. Although 17 new false positives were introduced in refinement step C to correct 53 model errors, the corrections were supported by experimental results. Aside from the *argD* case described above, we did not find any rule corrections that caused significantly more false positives for other knockout mutant or medium conditions. We further evaluated the effects the model changes had on predicted wildtype optimal metabolic flux distributions. Flux variability analysis was done before and after Refinement A with *i*JR904 and before and after Refinements B+C with *i*AF1260. This analysis was done for conditions in which the models predict non-zero growth rates before and after the refinements (84 media conditions for *i*JR904 and 112 media conditions for *i*AF1260), since the model changes were not intended to affect these conditions. We found that the model changes had no significant effect on the predicted wildtype fluxes for the 84 and 112 conditions examined (maximum and minimum predicted flux values changed by less than 0.004 mmol/gDW/hr, which corresponds to ∼0.04% of the carbon source uptake rates), except for the two conditions with L-malate and D,L-malate as carbon sources. In these two conditions, the regulatory rule change for *dctA* in Refinement C allows D-malate to be transported and L-malate to be transported with a more energetically efficient transporter. As a result higher growth rates can be achieved for these two conditions and the optimal flux distributions will change significantly.

### Use of *Gene*Force algorithm for predicting mechanisms for rescuing non-growth phenotypes

In addition to identifying regulatory rules that cause inconsistencies between model predictions and experimental growth phenotypes, another utility of the *Gene*Force algorithm is to identify genes whose transcriptional regulation prevents cells from growing. In this case the integrated model and regulatory rules are correct, and the un-expressed state of certain metabolic genes prevents the cells from utilizing a particular carbon or nitrogen source. The algorithm functions in the same manner as before, with the difference being that it is used in −/+/− cases in which cells are incapable of growing experimentally, the metabolic model indicates that the genes necessary to support growth are present in the genome, but the integrated metabolic and regulatory model correctly predicts a non-growth phenotype because the necessary genes are not expressed. While the algorithm would falsely violate regulatory rules in order to allow the model to achieve a non-zero growth rate, such false violations are of interest since they indicate which genes if over-expressed would allow for growth. Experimentally, such results could be tested by increasing the expression of the identified genes.

**Figure 5:**
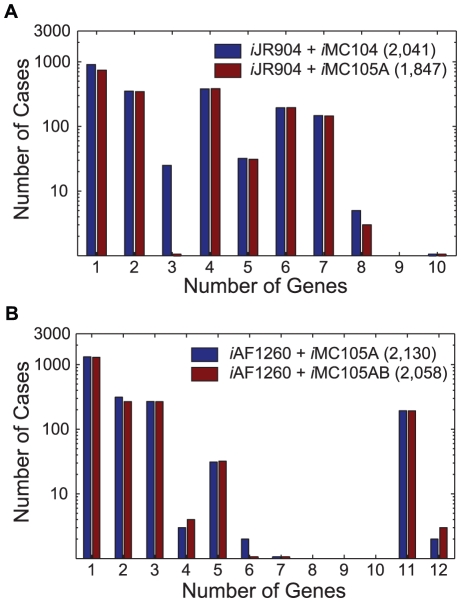
Number of Rule Corrections Needed to Rescue Non-Growth Phenotypes. Distribution of ‘rescue non-growth’ (−/+/−) cases before and after rule corrections for (**A**) *i*JR904 with rules from the *i*MC104 (with Lrp modified regulatory rules) and *i*MC105A, and (**B**) *i*AF1260 with rules from *i*MC105A and *i*MC105AB. The number in parenthesis in the legends indicates the total number of (−/+/−) cases for the different integrated models. For each −/+/− case on the minimum number of genes requiring regulatory rule violations was determined. Panels a and b are histograms representing the number of cases requiring 1, 2, 3, 4, 5, 6, 7, 8, 9, 10, 11, or 12 genes to be overexpressed to rescue non-growth phenotypes.

In our analysis of ∼32,000 mutant phenotypes using *i*JR904+*i*MC105A, we identified ten medium conditions, where the *Gene*Force algorithm repeatedly identified genes whose over-expression could enable aerobic growth of mutant (and likely wild-type) *E. coli* strains. In each of these nutritional states: (i) the majority of the *E. coli* knockout mutants were unable to grow, (ii) the metabolic model incorrectly predicts growth, and (iii) the integrated metabolic and regulatory model correctly predicts no growth. In seven out of the ten aerobic conditions, either a single gene or a single operon was needed to be expressed in violation of the regulatory rules to allow for growth. This list included *citT*, *xylA*, *allC*, *fucO*, *atoDAEB*, *ttdAB*, and *nirBD*, which correspond to the different medium conditions listed in Table 3. The distribution of the number of genes needing overexpression to rescue these non-growth phenotypes (−/+/−) occurred in the first two refinement steps (Supporting Information Table S1 and Supporting Information Table S2) is shown in Figure 5. Similar to the case for rule corrections (Figure 3), most of the rescue non-growth cases required over-expressing a single gene. The refinement of the regulatory rules (listed in Table 1) slightly reduced the number of rescue non-growth cases by ∼1–10% as some cases changed from −/+/− to −/+/+ (Figure 5).

**Table 3:** Single genes or operons that are predicted to rescue non-growth phenotypes under aerobic conditions.

Media	Gene	Condition
Citrate	*citT*	Carbon Source
Sucrose	*xylA*	Carbon Source
1,2 propanediol	*fucO*	Carbon Source
Butyrate	*atoDAEB*	Carbon Source
L-tartrate	*ttdAB*	Carbon Source
Allantoin	*allC*	Nitrogen Source
Nitrite	*nirBD*	Nitrogen Source

We subsequently looked for experimental evidence in the literature that would corroborate the algorithm's predictions of genes whose overexpression can rescue non-growth phenotypes. We found direct evidence in support of *citT*, *fucO* and *atoDAEB* rescuing the inability of wild-type *E. coli* to grow aerobically on citrate, 1,2-propanediol, and butyrate, respectively [34]–[38]. The *citT* gene encodes a citrate transporter, and Pos et al. have shown that plasmid mediated over-expression of *citT* allows for aerobic growth on citrate [34]. The 1,2-propanediol oxidoreductase (FucO), is required for growth on 1,2-propanediol anaerobically, but under aerobic conditions this gene is not expressed preventing utilization of this compound. Constitutive expression of *fucO* leads to an ability to grow on 1,2-propanediol aerobically [35], [37]. Wild-type *E. coli* is unable to utilize saturated short chain fatty acids, such as butyrate, and studies have shown that the constitutive expression of *atoC*, an activator of the *atoDAEB* operon, instills the ability to grow on butyrate [36], [38].

We were unable to find direct evidence in support of *allC* (allantoin) and *ttrAB* (L-tartrate) but these genes encode enzymes in the catabolic pathways for these substrates. Wild-type *E. coli* can utilize allantoin as a sole nitrogen source anaerobically, but not as a sole carbon source [39]. The inability to degrade allantoin aerobically is thought to be due to the oxygen mediated inhibition of the regulatory gene *allS*, which is an activator of the allantoin regulon containing *allC* [39]. It is possible that constitutive expression of *allC* would allow for utilization of allantoin in an oxic environment. Similar strategies may also be proposed for the *ttrAB* operon, which is also repressed in the presence of oxygen thereby preventing aerobic growth on L-tartrate, a substrate that can be used anaerobically [40].

### Conservation of transcriptional regulation in *E. coli* and *S. typhimurium*

The two bacterial strains, *S. typhimurium* LT2 and *E. coli* K-12 MG1655 are closely related and both organisms have been well studied experimentally and modeled. However, the transcriptional regulatory network of *S. typhimurium* is less characterized experimentally, than *E. coli*'s, and a genome-scale transcriptional regulatory model for *S. typhimurium* is not available. Recently, a metabolic network model, (*i*RR1083) for *S. typhimurium* was published [20], and we investigated the effects of conserving the *E. coli* transcriptional regulatory interactions in *S. typhimurium* by superimposing the *E. coli* regulatory constraints on the *Salmonella* metabolic network. The regulatory model *i*MC105A was integrated with the metabolic model *i*RR1083, and we evaluated whether this chimeric model was consistent with growth phenotypes for *S. typhimurium*. The expectation was that if the transcriptional regulatory networks were highly conserved few regulatory rule violations would be needed to correctly predict growth.

We transferred the regulatory rules in *i*MC105A for *E. coli* genes to their orthologs in *S. typhimurium*, and used the GPR association in the *S. typhimurium* metabolic network to constrain fluxes through the metabolic reactions. Among the 1,083 *S. typhimurium* genes included in *i*RR1083, 782 genes had orthologs in *E. coli* that were included in *i*JR904 (which included a total of 904 metabolic genes). Additionally, among the 105 *E. coli* transcription factors in *i*MC105A, we found 86 had orthologs in *S. typhimurium* which were incorporated into the chimeric model. These differences in conservation of metabolic and regulatory genes allowed us to transfer approximately 83% of the regulatory rules in *i*MC105A, while discarding the remaining rules associated with metabolic genes not present in *S. typhimurium* or regulatory rules involving transcription factors present only in *E. coli*. Any metabolic ortholog present in *S. typhimurium* but regulated by transcription factors without orthologs in *S. typhimurium* were kept unregulated in the chimeric model.

We applied the *Gene*Force algorithm to this hybrid *E. coli* regulatory-*S. typhimurium* metabolic model and evaluated model predictions against wild-type *S. typhimurium* growth phenotypes in 196 medium conditions (Supporting Information Table S5), which resulted in a surprisingly small number of regulatory rule violations, suggesting a highly conserved transcriptional regulatory network between *E. coli* and *S. typhimurium*, at least for conserved orthologs. As seen in Table 4, only a total of 18 genes (out of 505 genes with regulatory rules) needed regulatory rule corrections, some of which (*argD*, *rhaR* and *rhaS*) also needed rule corrections in subsequent refinements of the *E. coli* integrated model as well (Table 2, correction list B). Thus, 15 out of the 18 genes suggested some regulatory differences between the two species (Table 4). For example, the *prp* operon was forced to be active by the algorithm because *S. typhimurium* is capable of utilizing 1, 2-propanediol aerobically while *E. coli* is not [41]. The *prpBCDE* operon of *S. typhimurium* encodes enzymes that are needed for utilization of 1,2 propanediol [42]. Regulatory rules in *i*MC105A state that the *E. coli prp* operon is induced by propionate, while for *S. typhimurium*, there is evidence that this operon is induced by the coordinated function of regulatory proteins PrpR, IHF, and RpoN, where activation of PrpR is induced by 2-methylcitrate, a reaction intermediate in the 1,2-propanediol utilization pathway [43], [44]. Therefore, the algorithm correctly identified the *prp* genes as having incorrect rules for *S. typhimurium* but not for *E. coli*.

**Table 4:** Regulatory rules needing correction when integrated with a *S. typhimurium* metabolic network.

Gene	Original rule	Refined Rule
*prpB*	(ppa(e)>0)	(PrpR AND RpoN AND (HimA AND HimD))
*prpC*	(ppa(e)>0)	(PrpR AND RpoN AND (HimA AND HimD))
*prpD*	(ppa(e)>0)	(PrpR AND RpoN AND (HimA AND HimD))
*prpR* b		(MCITS>0)
*himA* b		ON
*himD* b		ON
*fadL*	((NOT (Crp OR FadR OR OmpR)))	ON
*fucO*	(((((FucR) OR (rmn(e)>0)) AND (NOT (o2(e)>0))) AND Crp) OR (((FucR) OR (rmn(e)>0)) AND (NOT (o2(e)>0))))	(fuc-L(e)>0 OR rmn(e)>0)
*glnA*	(Crp AND RpoN)	ON
*ttdA*	(NOT(o2(e)>0) AND (tartr-L(e)>0))	(tartr-L(e)>0)
*ttdB*	(NOT(o2(e)>0) AND (tartr-L(e)>0))	(tartr-L(e)>0)
*focA*	(ArcA OR Fnr AND (Crp OR NOT (NarL)))	ON
*argD ^a^*	(NOT ArgR)	(NOT ArgR) OR (arg-L(e)>0)
*prsA*	(NOT PurR)	ON
*guaA*	(NOT (PurR AND Crp))	ON
*guaB*	(NOT (PurR AND Crp))	ON
*rhaS ^a^*	(RhaR)	(RhaR OR (RhaR AND Crp))
*rhaR ^a^*	(rmn(e)>0)	(rmn(e)>0 OR lyx(e)>0 OR man(e))

Corrections common to *E. coli* and *S. typhimurium*.

b *prpR*, *himA* and *himD* were added to the regulatory network to update the regulatory rule for the *prpBCD* operon, and were not part of the original 505 regulatory rules for *S. typhimurium*.

The *glnA* gene (encoding glutamine synthetase) was also identified as requiring a rule correction in *S. typhimurium* but not in *E. coli* for growth on glucose and D-gluconate medium. This difference is primarily attributed to differences in the GPR association for glutamine synthetase between the two metabolic networks, where *glnA* encodes a sole enzyme for glutamine synthesis in *S. typhimurium*, whereas in the *E. coli* models an additional isozyme YcjK can catalyze the same reaction when *glnA* expression is suppressed. Recently, however the YcjK has been shown to be incapable of synthesizing glutamine from glutamate and ammonia so the regulatory rule for *glnA* and GPR association for glutamine synthetase needs to be updated in the *E. coli* model as well [45]. Similarly, the *focA* gene encodes the sole formate transporter in *S. typhimurium* but has an alternative gene *focB* in *E. coli*, which explains why the *focA* regulatory rule was not problematic in our analysis of *E. coli* growth phenotypes.

The initial integrated metabolic and regulatory model for *S. typhimurium*, when tested against the wild-type *S. typhimurium* growth phenotypic data in 196 medium conditions was 77% accurate. The unregulated metabolic model (unregulated *i*RR1083) was 82% accurate against the same growth phenotyping data. After introducing the refined rules (Table 4), the refined integrated metabolic and regulatory model was able to achieve 83% accuracy for this dataset, a value similar to those found in this study for integrated models of *E. coli* metabolism and regulation.

## Discussion

In this work we developed a new optimization-based approach, *Gene*Force, for systematically refining a genome-scale transcriptional regulatory model by comparing model predictions against high-throughput growth phenotypic data. The developed approach was used to (i) refine existing transcriptional regulatory and metabolic models of *E. coli* and suggest regulatory rule corrections, (ii) explain how transcriptional regulation prevents cellular growth in certain conditions and identify genes which can rescue non-growth phenotypes if expressed, and (iii) construct and refine a new integrated regulatory and metabolic model for *S. typhimurium*.

We showed that even well curated transcriptional regulatory and metabolic models for *E. coli* [3], [21], [22] can be further improved by using the developed approach. Here, cases where the integrated model under-predicted growth (cells grew experimentally and the metabolic model predicted growth, but the integrated model did not predict growth) were used to improve the integrated metabolic and regulatory model. A total of 42 model corrections (27 listed in Table 1 and an additional 15 described in the Materials and Methods section) were identified and when implemented they improved the accuracy of the models by 1–8%. The improved integrated metabolic and regulatory model predictions were found to better predict metabolic mutant phenotypes than other constraint-based methods using only metabolic models. When the *i*AF1260 metabolic model was used, flux balance analysis (FBA) was ∼76.5% accurate and minimization of metabolic adjustment (MOMA) [46] was ∼75.6% accurate (data not shown), while the integrated metabolic and regulatory model (*i*AF1260+*i*MC105ABC) was ∼79.6% accurate when predictions were made for the metabolic gene knockouts. The integration of metabolic and regulatory network models is thus important for being able to more accurately predict behavior of metabolic mutants, as well as, transcription factor mutants.

In addition to fixing incorrect model predictions, we showed that *Gene*Force can also be used to evaluate correct model predictions of non-growth conditions to explain how regulation prevents the use of particular nutrients since the needed enzymes are encoded in the genome. We used the approach to suggest a set of genes which if expressed can rescue non-growth phenotypes of mutant and wildtype strains. Experimental testing of these hypotheses would validate that particular metabolic transformations occur and could be used to engineer novel growth phenotypes in an organism.

In addition to applying the *Gene*Force approach to already developed and refined metabolic and regulatory *E. coli* models, we also applied it to a new integrated model for *S. typhimurium*. We constructed an initial transcriptional regulatory model for *S. typhimurium*, by transferring the regulatory network from a closely related organism. We then applied the approach to correct regulatory rules in the integrated metabolic and regulatory model of *S. typhimurium* [20]. The results showed that the transcriptional regulatory network in *E. coli* is highly consistent with the growth phenotypes of *S. typhimurium*, indicating that the regulatory networks in these two organisms may be highly conserved between these two organisms. A similar observation was found previously by Babu et al., where ∼90% of *E. coli* regulatory interactions were predicted to be conserved in *S. typhimurium* based on the presence of orthologs of transcription factors and their target genes in *S. typhimurium* [47]. While the resulting integrated model is still consistent with observed phenotypes for *S. typhimurium*, additional work is still needed to expand this initial regulatory model to include organism-specific regulatory interactions (such as altered regulons and regulons that are unique for *S. typhimurium*).

The number of available metabolic reconstructions is rapidly increasing, which is made possible by the increased number of genome sequences [48]. However, the development of genome-scale transcriptional regulatory models is currently limited by the lack of available data for most organisms. Different approaches have been developed to model transcriptional regulatory networks (reviewed in [10]), but a Boolean approach has been commonly used for building genome-scale transcriptional regulatory models due to its scalability. Integrated models of metabolism and transcriptional regulation have been developed using a Boolean approach for some model organisms, such as *E. coli* [3] and *S. cerevisiae* [4], but our current understanding of transcriptional regulation in microorganisms is still limited due to its complexity, interconnectivity, and intrinsic noise in these networks compared to metabolism. The *Gene*Force approach can be very useful for validating and refining transcriptional regulatory models against new experimental data, as well as for developing new regulatory models where initial models often yield a number of predictions that are inconsistent with experimental data. In the past, the identification of regulatory rules causing inconsistencies between model predictions and experimental observations was done through a time intensive, trial and error process [3].

Other types of non-Boolean methods are needed to integrate genome-scale metabolic and regulatory models, since Boolean approaches cannot capture all transcriptional regulatory interactions (e.g. regulation of essential genes) and gene expression and metabolic fluxes have variable levels that cannot be reflected using ‘On/Off’ variables. Modeling methods are available to predict gene expression levels [49], and these predictions could be used to constrain metabolic fluxes [50]–[52] at a genome-scale. The *Gene*Force algorithm could be easily extended to consider other types of integrated metabolic and regulatory models as they are developed, where the number of genes needing expression levels higher than those predicted by the regulatory models could be minimized. As such, the approach would still reconcile integrated metabolic and regulatory network models with observed growth phenotypes and suggest improvements of such models. Other approaches have been developed for metabolic models that use experimentally determined flux distributions as a means to refine metabolic models [53], and the *Gene*Force algorithm could be extended to compare more quantitative data including biomass yields (where the measured yields are used to determine the minimum growth rate threshold) and measured fluxes (where model fluxes are constrained to be a certain distance from the experimental values) as such quantitative data become available at a large-scale.

Although automated approaches for refining metabolic models have been developed [7], [9], [54], [55], such an approach has not been created for integrated models of metabolism and transcriptional regulation. The approach developed here finds a minimum set of refinements needed to correct one case at a time. While we did not find it to be a significant problem here, it is possible that making model refinements to correct one case may cause a significant number of new incorrect predictions for other cases. Approaches that consider multiple cases simultaneously could be advantageous, but they were not considered here because of the added computational burden for considering all conditions simultaneously. The approach described here can be used to improve transcriptional regulatory network models by accounting for how a hypothesized regulatory network will affect metabolism and thereby cellular behavior. We envision that predictions of *cis*-regulatory networks, based on genomic analysis and/or experimental data, can be translated into Boolean regulatory models that can be rapidly refined using our developed approach. The identified refinements can then suggest further experiments and lead to a re-evaluation of *cis*-regulatory networks. By integrating models of metabolism and regulation, phenotypic data can be evaluated against regulatory network predictions (which is difficult to do without a metabolic model), thereby expanding the types of datasets (e.g. gene expression, genome sequence, and DNA-protein interaction) that can be used to reconstruct transcriptional regulatory networks.

## Materials and Methods

### Strains

The Keio collection of in-frame single-gene deletion strains [25] and *E. coli* K-12 BW25113 (the parent strain of the Keio collection) were used to confirm the model changes identified by *Gene*Force. The kanamycin resistant gene (*kan*) was removed from the single-deletion strains before screening mutant phenotypes in the microplate reader (for methods see [56]). In addition, seven double mutants (*lrp*::*kan* Δ*ilvB*, *lrp*::*kan* Δ*ilvN*, *lrp*::*kan* Δ*ilvH*, *lrp*::*kan* Δ*ilvI*, *lrp*::*kan* Δ*dctA*, *rpiA*::*kan* Δ*rpiB*, and *cycA*::*kan* Δ*dsdX*) were generated using P1 transduction (for methods see [57]).

### Growth phenotyping experiments

Phenotype microarray (PM) experiments were conducted for the *arcA::kan*, *purR::kan*, and *lrp::kan* strains from the Keio collection using PM1 and PM2 plates following manufacturer protocols (Biolog Inc., CA). Briefly, strains were grown on BUG+B agar plates and resuspended in inoculating fluid containing Dye A and loaded onto plates. Plates were incubated at 30°C and absorbance readings were taken at 600nm at 24 and 48 hours. Other strains were screened for growth in triplicate at 37°C in a Tecan Infinite 200 microplate reader (Tecan Group Ltd., Switzerland). Optical density measurements at 600 nm were taken by the microplate reader every 15 minutes. The Tecan OD measurements (OD_Tecan_)were converted to an OD value in a spectrophotometer with a 1cm pathlength (OD_600_), using a predetermined linear relationship, OD_600_ = (2.566)OD_Tecan_+0.0028. Strains were pre-cultured overnight in 2 g/liter glucose-supplemented M9 minimal medium, except for a few strains (listed in Figure 4A) that were evaluated for their ability to grow on glucose which were instead pre-cultured in LB medium. Pre-cultured cells were washed and resuspended in media containing a new carbon source so that the starting OD (at 600 nm) was around 0.05. All carbon sources were tested in M9 minimal medium (6.8 g of Na_2_HPO_4_, 3 g of KH_2_PO_4_, 0.5 g of NaCl, 1 g of NH_4_Cl, 2 ml of 1M MgSO_4_, and 100 µl of 1 M CaCl_2_ per liter) supplemented with 2 g/liter of carbon source.

### Data analysis

High-throughput growth phenotyping (Biolog Inc., CA) data for *E. coli* from the ASAP database [18] were analyzed to assign “growth (+)” or “no growth (−)” for mutants grown in different conditions. In addition to the dataset (Mutant Biolog Data I) evaluated by Covert and colleagues [3], an additional dataset (Mutant Biolog Data II) was analyzed in this study. We considered the phenotype microarray (PM) data for carbon (PM1 and PM2) and nitrogen sources (PM3) that can be simulated by the computational models, which consisted of 223 mutants in 130 conditions or 303 mutants in 153 conditions, depending on which model was used (see below). For each PM plate, the negative control value was subtracted from each data point (OD600), and a cutoff parameter of 0.1 was applied to determine whether the cells could grow (+) or not grow (−). The cutoff parameter was obtained by separating a bimodal-like distribution of the data (Supporting Information Figure S1), and the results were not highly sensitive to this parameter. Another set of high-throughput phenotyping data for single gene knockout mutants of *E. coli* [19] was also used. This dataset includes growth phenotypes for 1,440 mutants in 95 environmental conditions using a GN2-MicroPlate (Biolog Inc., CA). However, the conditions that can be simulated by the models consist of only 102 mutants in 30 conditions or 128 mutants in 31 conditions depending on the model used, since the majority of evaluated mutants involved knockouts of genes with unknown function. The phenotypic data for three global transcription factor knockout mutants (Δ*arcA*, Δ*purR*, and Δ*lrp*) was generated in this study using the phenotype microarrays (Biolog Inc., CA) as described above. In this study, we have excluded the phenotypic data for cells grown on formate and L-serine as carbon sources, and xanthine and xanthosine as nitrogen sources, as they are likely false positives in the PM datasets (formate [3]; xanthine and xanthosine [7] and L-serine (tested in this study, data not shown) ).

### Models and simulation conditions

The genome-scale models of metabolism (*i*JR904 [21], *i*AF1260 [22]) and regulation (*i*MC104^v1^ [3]) for *E. coli* were integrated and used in this study. First, regulatory interactions for the global transcription factor, Lrp, were updated in the regulatory rules represented in the *i*MC104 model based on the recent regulatory reconstruction from analysis of gene expression and ChIP-chip data [17]. The Lrp reconstruction categorized regulatory interactions into six different modes based on the gene expression responses of genes controlled by Lrp to exogenous leucine. We have converted each regulatory mode into Boolean logic rules, and updated the regulatory rules in conjunction with existing rules. Preliminary computational analysis was performed to identify essential genes for growth in glucose minimal media that were predicted to be un-expressed based on the updated Lrp rules; the regulatory rules for these seven essential genes were then changed back to the original ones before mutant phenotypes were evaluated. In addition, when the metabolic part of the integrated model was replaced with the recent metabolic reconstruction, *i*AF1260 instead of *i*JR904, another set of preliminary rule corrections were needed for the eight genes that are essential only in *i*AF1260, due primarily to changes in the biomass equation. These fifteen preliminary rule corrections were made before the integrated model was compared to mutant phenotypes, and thus they are not listed in Table 1 (see Supporting Information Table S6 for details).

Simulation conditions for the models were determined based on the available carbon or nitrogen sources in the media as previously described elsewhere [3] (see Supporting Information Table S7). When testing the growth on different carbon sources, ammonia was used as a nitrogen source and the maximum uptake rate for ammonia was constrained to be 10 mmol/gDW/hr. Pyruvate was used as a carbon source for testing growth on different nitrogen sources, and its uptake rate was constrained to be 11.3 mmol/gDW/hr. Oxygen uptake rate was constrained to be 10 mmol/gDW/hr for all cases, and uptake rates for other essential nutrients in each model were specified as listed in Supporting Information Table S7.

### FBA and steady-state regulatory flux balance analysis (SR-FBA)

Flux balance analysis (FBA) [58] was performed to predict the maximum growth rate for mutants under different conditions using the metabolic models. In order to simulate gene deletions in the metabolic models, we have included GPR associations where reactions are constrained to have zero flux if an associated gene is deleted. For the integrated metabolic and regulatory models, we have systematically formulated an SR-FBA problem [15] with gene knockout and transcriptional regulatory constraints [16]. Predictions were made by maximizing growth rate for each mutant in each condition. If the maximum growth rate was positive then the model predicted growth is designated as (+), or otherwise designated as (−).

### *Gene*Force formulation

*Gene*Force identifies the minimal set of genes that are required for growth, but are unexpressed in a given condition due to transcriptional regulatory constraints. In the *Gene*Force formulation, unexpressed genes are allowed to violate the regulatory rules, and the number of violations is minimized to prevent unnecessary rule violations. A rule violation is implemented by introducing surrogate gene expression indicator variables (y′_g_) to allow flux through reactions whose associated genes are not expressed according to the Boolean regulatory rules. A minimum growth rate requirement is introduced by setting the lower bound for growth rate to a minimum threshold value, and the threshold value was set to 10% of the maximum growth rate predicted by the metabolic model in this study. The algorithm was relatively insensitive to threshold values between 5 and 50% (see Supporting Information Table S8), because most integrated model growth rate predictions were above 80% or below 5% of the metabolic model predicted growth rate (see Supporting Information Figure S2). Alternative optimal solutions were found by adding integer-cut constraints and re-solving the problem. See Supporting Information Text S1 for more details.

### Rule correction

To identify possible regulatory rule corrections, we analyzed the cases where the metabolic model and experimental data agree that the mutant can grow, but the integrated model predicts no growth (+/+/−; corresponding to experimental data / metabolic model / integrated model). If a certain set of regulatory rules were repeatedly violated in the *Gene*Force solutions to allow for growth of a specific mutant or in a particular medium condition, the regulatory rules for those genes were corrected based on experimental evidence from the literature. When alternative optimal solutions were available, meaning that different sets of rule violations could correct the non-growth phenotype predictions, we examined each set of solutions and chose the most appropriate one for the specific case based on results from additional experiments and/or information in the literature. If a set of rule corrections caused inconsistencies in other mutant or medium condition, such corrections were not made unless there was strong experimental evidence for the rule correction.

## Supporting Information

Figure S1 Histograms of OD600 measurement in Biolog Phenotype Microarrays (PM).
(0.19 MB PDF)

Figure S2 Histograms of relative growth rate predictions by *i*JR904 and *i*MC104 (with Lrp modified regulatory rules) for postive and negative experimental growth phenotypes.
(0.07 MB PDF)

Table S1 Phenotype-model comparison using *i*JR904 and *i*MC104 (with Lrp modified regulatory rules).
(0.51 MB XLS)

Table S2 Phenotype-model comparison using *i*AF1260 and *i*MC105A.
(0.77 MB XLS)

Table S3 Phenotype-model comparison using *i*AF1260 and *i*MC105AB/*i*MC105ABC for Δ*arcA*, Δ*purR*, and Δ*lrp* mutants.
(0.03 MB XLS)

Table S4 Detailed statistics of model-data comparisons and alternative optimal solutions for each refinement step.
(0.03 MB XLS)

Table S5 Phenotype-model comparison using *i*RR1083 (*Salmonella typhimurium* LT2) and *i*MC105A.
(0.03 MB XLS)

Table S6 Preliminary analysis for integrating the new Lrp reconstruction and metabolic model *i*AF1260.
(0.05 MB XLS)

Table S7 Simulation conditions for Biolog Phenotype Microarrays (PM) and GN2-Microplate.
(0.06 MB XLS)

Table S8 Sensitivity analysis for different values of minimum growth rate requirement using *i*JR904 and *i*MC104 (with Lrp modified regulatory rules).
(0.03 MB XLS)

Text S1 Detailed description of the *Gene*Force formulation.
(0.05 MB DOC)
