# Supplementary material for: An Automated Phenotype-Driven Approach (GeneForce) for Refining Metabolic and Regulatory Models
Source: PLoS Comput Biol. 2010 Oct 28;6(10):e1000970. doi: 10.1371/journal.pcbi.1000970 (PMC2965739; doi:10.1371/journal.pcbi.1000970)
Supplement: Figure S1 — Histograms of OD600 measurement in Biolog Phenotype Microarrays (PM). (0.19 MB PDF) [file pcbi.1000970.s001.pdf]

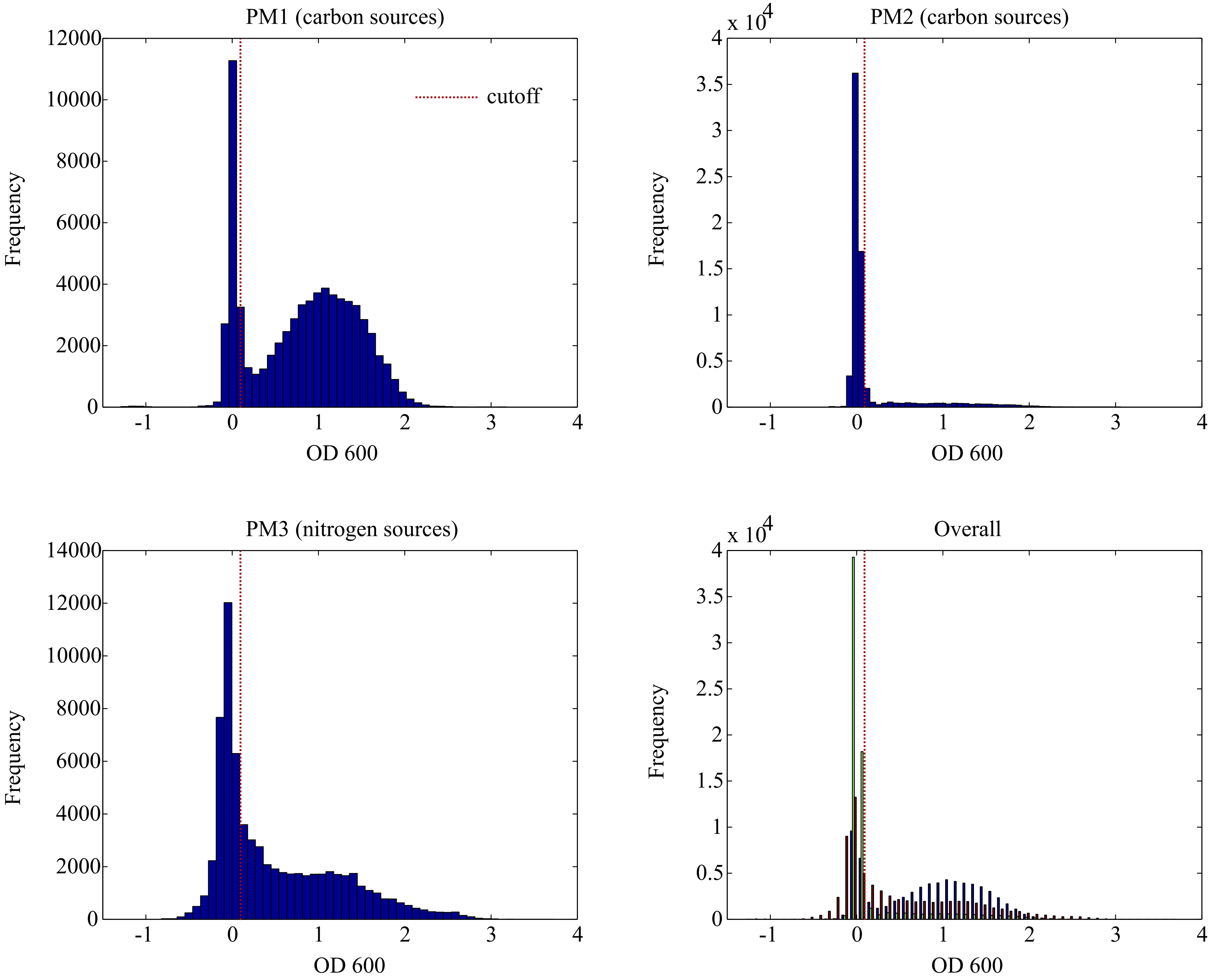

Supporting Information Figure S1: Histograms of OD600 measurement in Biolog Phenotype Microarrays (PM).
