# Supplementary figures and images for: An Automated Phenotype-Driven Approach (GeneForce) for Refining Metabolic and Regulatory Models
Source: PLoS Comput Biol. 2010 Oct 28;6(10):e1000970. doi: 10.1371/journal.pcbi.1000970 (PMC2965739; doi:10.1371/journal.pcbi.1000970)

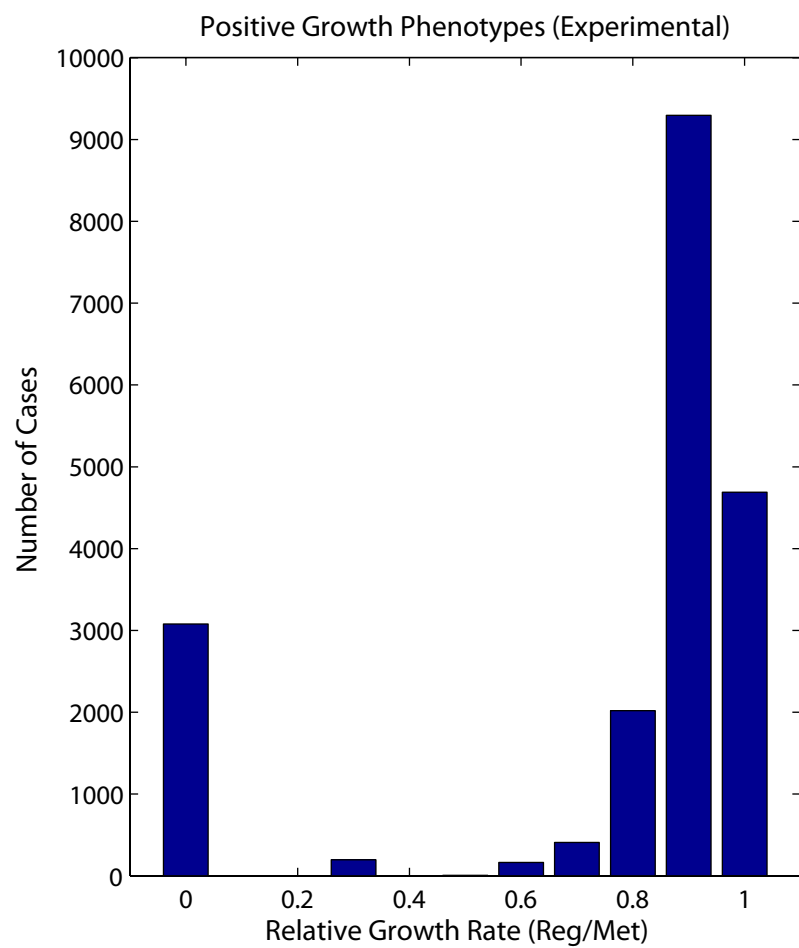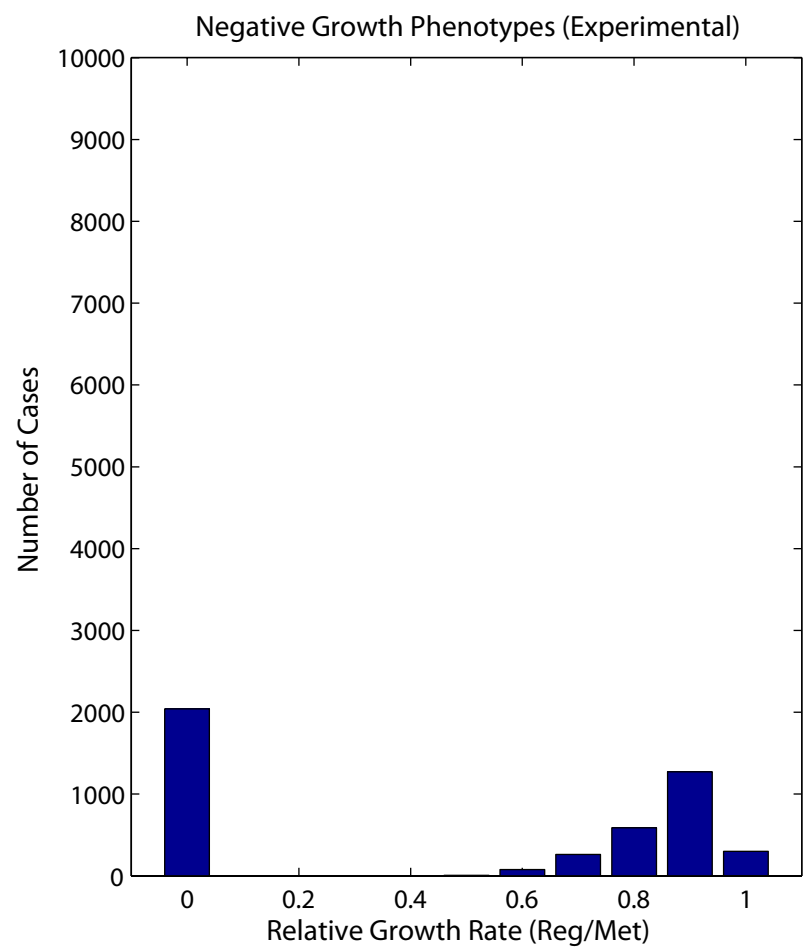

Supplement: Figure S2 — Histograms of relative growth rate predictions by iJR904 and iMC104 (with Lrp modified regulatory rules) for postive and negative experimental growth phenotypes. (0.07 MB PDF) [file pcbi.1000970.s002.pdf]
