# Supplementary material for: An Automated Phenotype-Driven Approach (GeneForce) for Refining Metabolic and Regulatory Models
Source: PLoS Comput Biol. 2010 Oct 28;6(10):e1000970. doi: 10.1371/journal.pcbi.1000970 (PMC2965739; doi:10.1371/journal.pcbi.1000970)
Supplement: Text S1 — Detailed description of the GeneForce formulation. (0.05 MB DOC) [file pcbi.1000970.s011.doc]

**GPR association and transcriptional regulatory constraints**: GPR associations and transcrip­tional regulatory rules were systematically formulated as linear constraints with binary variables (Kim and Reed, submitted). Each reaction, j, with a known GPR can be carried out by the set of associated enzyme complex(es), n(j). Two binary variables b_n_ and d_j_ indicate whether the enzymes are present (b_n_ = 1) and whether the reaction can occur (d_j_ = 1), respectively. If any of the enzymes for reaction j is present (any b_n(j)_ = 1), then the reaction can have a non-zero flux (d_j_ = 1) as constrained by equation (1) and (2). If the reaction can occur, the flux value of each reaction is constrained by its lower bound (v_j,lb_) and upper bound (v_j,ub_). If all the enzymes are not present (all b_n(j)_ = 0), then the reaction cannot occur (d_j_ = 0) by equation (3).

*d_j_ v_j,lb_ ≤ v_j_ ≤ d_j_ v_j,ub_ (1)*

*d_j_ ≥ b_n(j)_ (2)*

*d_j_ ≤ ∑_n(j)_ b_n(j)_ (3)*

Each enzyme complex, n, is associated to a set of genes, g(n). If all of the associated genes for enzyme n are expressed (all y_g(n)_ = 1), then the enzyme is present (b_n_ = 1) as determined by equation (4). If any of the enzyme subunits are not expressed (any y_g(n)_ = 0), the enzyme is not present (b_n_ = 0) as determined by equation (5).

*(b_n_ – 1) ≥ ∑_g(n)_ (y_g(n)_ – 1) (4)*

*b_n_ ≤ y_g(n)_ (5)*

Each gene, g, is transcribed if the conditions for its expression, m(g), are satisfied. If any of the conditions for gene g expression is satisfied (any a_m(g)_ = 1), the corresponding gene is expressed (y_g_ = 1) using equation (6). If all the conditions for expression are not satisfied (all a_m(g)_ = 0), then the gene is not expressed (y_g_ = 0) as determined by equation (7). Similarly, each transcription factor, TF, is active (x_TF_ = 1) if any of the conditions for its activity is satisfied (any a_m(TF)_ = 1) using equation (8). If all the conditions for activity are not satisfied (all a_m(TF)_ = 0), then the transcription factor is inactive (x_TF_ = 0) as determined by equation (9).

*y_g_ ≥ a_m(g)_ (6)*

*y_g_ ≤ ∑_m(g)_ a_m(g)_ (7)*

*x_TF_ ≥ a_m(TF)_ (8)*

*x_TF_ ≤ ∑_m(TF)_ a_m(TF)_ (9)*

Each condition for gene expression or transcription factor activity, m, has its associated effectors, r(m). If all the associated activators are active and all of the associated repressors are inactive ((all x^Act^_r(m)_ = 1) AND (all x^Rep^_r(m)_ = 0)), then the condition for gene expression or transcription factor activity is satisfied (a_m_ = 1) as determined by equation (10). If any of the activators are inactive or any of the repressors are active ((any x^Act^_r(m)_ = 0) OR (any x^Rep^_r(m)_ = 1)), the condition for gene expression or transcription factor activity is not satisfied (a_m_ = 0) using equations (11) and (12).

*(1 – a_m_) ≤ ∑_r(m)_ ((1 – x ^Act^_r(m)_) + x ^Rep^_r(m)_) (10)*

*a_m_ ≤ x ^Act^_r(m)_ (11)*

*a_m_ ≤ (1 – x ^Rep^_r(m)_) (12)*

Each effector, r, can be a transcription factor (TF), environmental stimuli (ES), positive metabolic ﬂux (PF), or negative metabolic flux (NF). Intracellular and extracellular stimuli were reflected by the positive or negative metabolic flux indicators by equations (13) – (16), where intracellular stimuli are dependent on the flux values of internal reactions and extracellular stimuli are dependent on the flux values of exchange reactions (secretion or uptake). A threshold value (ε = 10^-3^) is used to determine the positive (v_PF_ *≥* ε) or negative flux (v_NF_ *≤* –ε). Other environmental stimuli such as oxidative stress or high osmolarity were assumed to be absent in this study as indicated by equation (17).

*v_PF_ ≤ (v_PF,ub_ – ε) x_PF_ + ε (13)*

*v_PF_ ≥ (v_PF,lb_ – ε)(1 – x_PF_) + ε (14)*

*v_NF_ ≤ (v_NF,ub_ + ε)(1 – x_NF_) – ε (15)*

*v_NF_ ≥ (v_NF,lb_ + ε) x_NF_ – ε (16)*

*x_ES_ = 0 (17)*

**FBA and steady-state regulatory flux balance analysis (SR-FBA):** Flux balance analysis (Pri*ce et* al, 2004) was performed to predict the maximum growth rate for mutants under different conditions using the metabolic model. Predictions by the metabolic models were made by maximizing growth rate (v_Biomass_) for each mutant in each condition (where reactions are constrained to have zero flux if an associated gene is deleted). If the maximum v_Biomass_ was positive then the model predicted growth is designated as (+), and if the maximum v_Biomass_ was zero then the model predicted growth is designated as (-). In order to predict the maximum growth rate using the integrated metabolic and regulatory models, we have systematically formulated an SR-FBA problem (Shlo*mi et* al, 2007) with gene knockout constraints as the following.

*max v_biomass_*

*s.t S∙v = 0 : Steady-state Mass Balance (18)*

*y'_g_ = 0 if gene g is deleted (19)*

*y'_g_ = y_g_ if gene g is not deleted (20)*

*(b_n_ – 1) ≥ ∑_g(n)_ (y'_g(n)_ – 1) (21)*

*b_n_ ≤ y'_g(n)_ (22)*

*Equations (1)-(3), (6)-(17)*

*d_j_, b_m_, y'_g_, y_g_, a_n_, x_r_ ; {0,1}*

A gene knockout is implemented by introducing surrogate gene expression indicators (y'_g_) which can be independent of the gene expression indicators (y_g_). This formulation allows a gene to be deleted, and thus unexpressed (y'_g_= 0), even when its transcriptional regulatory constraints for expression are satisfied (y_g_ = 1). If a gene is not deleted, its surrogate gene expression indicator is equal to the gene expression indicator as determined in equation (20). Then, instead of the gene expression indicators, the surrogate gene expression indicators are used to determine enzyme presence using equations (21) and (22) instead of equations (4) and (5).

***Gene*Force formulation:** *Gene*Force identifies the minimal set of genes that are required for growth, but are unexpressed (y_g_ = 0) in a given condition due to transcriptional regulatory constraints (equations (6) – (17)). In the *Gene*Force formulation, genes are allowed to violate the regulatory rules if required for growth, and the number of violations is minimized to prevent unnecessary rule violations. A rule violation is implemented by introducing rule violation indicators (w_g_) along with surrogate gene expression indicators (y'_g_). In equation (23), the surrogate gene expression indicators are constrained to be greater than or equal to the gene expression indicators (y_g_), which allows un-expressed genes (y_g_ = 0) to become expressed (y'_g_ = 1). Thus, the rule violation indicator assumes a value of 1 only if the regulatory rules are violated because the gene is needed for growth. In the *Gene*Force formulation, the rule violation equation (23) replaces equation (20) in the SR-FBA formulation.

*w_g_ = y'_g_ – y_g_ ≥ 0 if gene g is not deleted (23)*

A minimum growth rate requirement is introduced by setting the lower bound for growth rate to a minimum threshold value, μthreshold (in this study μthreshold was set to 10% of the maximum growth rate predicted by the metabolic model). Other threshold values were tested (See Supplementary Table S8) but the algorithm appeared to be relatively insensitive to the threshold over a wide range of values (5-50% of the metabolic model predicted maximum growth). This is because most integrated model growth rate predictions were either above 80% or below 5% of the metabolic model predicted growth rate (see Supplementary Figure 2). Integer-cut constraints are added based on previously identified solutions (k) to generate alternative optimal solutions.

*v_biomass_ ≥ μ_threshold_ (24)*

*∑_g_W_g_^k^w_g_+1 ≤ ∑_g_W_g_^k^ (25)*

The objective function in *Gene*Force is to minimize the sum of rule violation indicators (min ∑_g_ w_g_), and the complete formulation is the following.

*min ∑_g_ w_g_*

*s.t Constraints (1)-(3), (6)-(19), (21)-(25)*

*w_g_, d_j_, b_m_, y'_g_, y_g_, a_n_, x_r_ ; {0,1}*
